# High Prevalence of Extended-Spectrum Beta Lactamases among *Salmonella enterica* Typhimurium Isolates from Pediatric Patients with Diarrhea in China

**DOI:** 10.1371/journal.pone.0016801

**Published:** 2011-03-01

**Authors:** Fangyou Yu, Qiang Chen, Xiaojun Yu, Qiaoqiao Li, Baixing Ding, Lehe Yang, Cong Chen, Zhiqiang Qin, Chris Parsons, Xueqing Zhang, Jinwei Huang, Yun Luo, Liangxing Wang, Jingye Pan

**Affiliations:** 1 Department of Laboratory Medicine, The First Affiliated Hospital of Wenzhou Medical College, Wenzhou, China; 2 Department of Laboratory Medicine, Jiangxi Provincial Children Hospital, Nanchang, China; 3 Department of Respiratory Medicine, The First Affiliated Hospital of Wenzhou Medical College, Wenzhou, China; 4 Department of Intensive Care Unit, The First Affiliated Hospital of Wenzhou Medical College, Wenzhou, China; 5 Division of Infectious Diseases, Department of Medicine, Medical University of South Carolina, Charleston, South Carolina, United States of America; 6 Department of Laboratory Medicine, Lishui Center Hospital, Lishui, China; 7 Department of Microbiology, Zhejiang Provincial Center for Disease Control and Prevention, Hangzhou, China; Charité-University Medicine Berlin, Germany

## Abstract

We investigated the extended-spectrum beta lactamases among 62 *Salmonella enterica* Typhimurium isolates recovered from children with diarrhea in a Chinese pediatric hospital. A large proportion of *S. enterica* Typhimurium isolates were resistant to multiple antimicrobial agents, including ampicillin (90.3%), tetracycline (80.6%), trimethoprim/sulfamethoxazole (74.2%), chloramphenicol (66.1%), cefotaxime (27.4%). Forty-nine (79.0%) of *S. enterica* Typhimurium isolates were positive for *bla*_TEM-1b_ and resistant to ampicillin. Thirteen *S. enterica* Typhimurium isolates (21.0%) were positive for *bla*_CTX-M-1-group_ and *bla*_CTX-M-9-group_, and all isolates harboring *bla*_CTX-M_ genes were positive for *IS*Ecp1. Two main clones (PFGE type A and D) accounted for nearly 70% of *S. enterica* Typhimurium isolates, and 7 CTX-M-producing isolates belonged to PFGE type D. Collectively, our data reveal multi-drug resistance and a high prevalence of extended spectrum beta lactamases among *S. enterica* Typhimurium isolates from children in China. In addition, we report the first identification of *bla*_CTX-M-55_ within *Salmonella spp*. Our data also suggest that clonal spread is responsible for the dissemination of *S. enterica* Typhimurium isolates.

## Introduction

Nontyphoidal *Salmonella* species, particularly *Salmonella enterica* Typhimurium, are the most common bacterial pathogens causing enteric infections among pediatric patients [1]. Antimicrobial agents are required for treating invasive infections caused by *Salmonella spp.*, and third-generation cephalosporins are commonly used to treat invasive infections or severe diarrhea because of their pharmacodynamic properties and the low prevalence of resistance [2]. However, increasing resistance to cephalosporins has been reported worldwide for *Salmonella spp.*, particularly *S. enterica* Typhimurium [3], [4], [5], [6], [7]. Resistance to broad-spectrum cephalosporins is often due to production of various plasmid-mediated ß-lactamases, especially CTX-M-type extended-spectrum β-lactamases (ESBLs) [4], [7], [8], [9]. Cefotaximases (CTX-M), comprised of five major CTX-M groups (1, 2, 8, 9, and 25), are associated with higher levels of hydrolytic activity against cefotaxime relative to ceftazidime and are distributed among a wide range of bacteria with clinical significance over a wide geographic area [10].

In China, a low prevalence of resistance to third-generation cephalosporins has been reported among *Salmonella spp.*, especially those isolated from pediatric patients [11], [12]. The aim of the present study was to investigate the frequency of antimicrobial resistance and cephalosporin resistance genes within *S. enterica* isolates from pediatric inpatients with diarrhea in a Chinese pediatric hospital. However, high prevalence of broad spectrum cephalosporin resistance and ESBLs were found among *S. enterica* Typhimurium isolates from children in China.

## Materials and Methods

### Bacterial isolates

From May 2007 to March 2009, a total of 62 *S. enterica* Typhimurium isolates were collected from stool samples collected from 62 inpatients aged ranging from 39 days to five years old with diarrhea in Jiangxi provincial pediatric hospital in Nanchang, China. *S. enterica* Typhimurium isolates were identified using standard biochemical tests and commercial typing antiserum according to the manufacturer's instructions. O and H antigens were characterized by slide agglutination with hyperimmune sera (Chengdu Biotech, China) and the serotype was assigned according to the Kauffmann-White scheme. Among 62 inpatients included, 34 and 28 patients were male and female. The ages of the patients were as follows: 39 days −1 year old, 37 patients; 1–2 years old, 10 patients; 2–3 years old, 5 patients; 3–4 years old, 6 patients and 4–5 years old, 4 patients. The antimicrobial agents used for treating the infections by *S. enterica* Typhimurium included penicillin, azithromycin, amoxicillin/sulbactam, latamoxef, cefoperazone/sulbactam, ceftazidime, ampicillin/sulbactam, ceftriaxone, cefazolin, ceftizoxime, and imipenem. Of 62 patients with diarrhea, 7, 4, 4 and 2 patients suffered bronchitis, pharyngitis, acute tonsillitis and meningitis, respectively. After treated by antimicrobial agents, all patients were cured and left hospital. Persistance of diarrhea ranged from 7 to 40 days. Addition to *S. enterica* Typhimurium, *Campylobacter spp*, *enteropathogenic Escherichia coli* and *Shigella spp* were not isolated from the stools of the patients included. This study focused on bacterial, so ethics approval was not needed according to the Ethics Committee of Wenzhou Medical College's regulatioans.

### Antimicrobial susceptibility

Antimicrobial susceptibility was determined by the disk diffusion method with 18 antimicrobial agents according to the criteria recommended by the CLSI [13], including including cefotaxime (30 µg), ceftazidime (30 µg), ampicillin (10 µg), aztreonam (30 µg), cefaclor (30 µg), cefoxitin (30 µg), piperacillin plus tazobactam (100/10 µg), imipenem (10 µg), meropenem (10 µg), chloramphenicol (30 µg), trimethoprim/sulfamethoxazole (1.25/23.75 µg), tobramycin (10 µg), gentamicin (10 µg), amikacin (30 µg), tetracycline (30 µg), nalidixic acid (30 µg), ciprofloxacin (5 µg), and levofloxacin (5 µg). MICs for cefotaxime and ceftazidime, were further determined by the agar dilution method in accordance with CLSI guidelines [13]. *Escherichia coli* ATCC 25922 was used as quality control strain for antimicrobial susceptibility testing.

### Detection of resistance genes

Total DNA was extracted by boiling. Briefly, a fresh bacterial colony was suspended in 150 µL of sterile distilled water and boiled at 100°C for 10 minutes. After centrifugation at 15000 rpm for 15 minutes at 4°C, the supernatant was removed and stored at −20°C for PCR assays. PCR and DNA sequencing were performed for the detection of ESBL genes in all ampicillin-resistant isolates with oligonucleotide primers previously described, including those for *bla*_TEM_, *bla*_SHV_, *bla*_CTX-M_, *bla*_VEB_ and *bla*_PER._genes [14], [15]. All amplicon sequences were compared with those in the GenBank nucleotide database (www.ncbi.nlm.nih.gov/blast/). PCR mapping experiments using combinations of the *ISEcp1* forward primers and the *bla*_CTX-M-1-group_ or *bla*_CTX-M-9 group_ reverse primers were performed to detect the flanking regions of *bla*_CTX-M-1-group_ and *bla*_CTX-M-9-group_ genes.

### Transfer of resistance genes

In order to determine whether cephalosporin resistance was transferable in *S. enterica* Typhimurium isolates, a conjugation experiment was carried out in Luria-Bertani broth with *E. coli* J53 as the recipient as previously described [16]. Transconjugants were selected on tryptic soy agar plates containing sodium azide (100 µg/mL) for counterselection and cefotaxime (30 µg/mL) for plasmid-mediated cephalosporin resistance selection.

### Pulsed-field gel electrophoresis (PFGE)

Chromosomal DNA was prepared from all *S. enterica* Typhimurium isolates and cleaved with 40 U *Xba*I. Electrophoresis was performed on 1% agarose gels in 0.5 M Tris/borate/EDTA buffer on a CHEF-Mapper XA PFGE system (Bio-Rad, Hercules, CA) for 22 h at 14°C, with run conditions of 6 V/cm, a pulse angle of 120° and pulse times from 5 to 20 s. A λDNA ladder (Amersham Biosciences) was used to confirm molecular mass and bands stained with ethidium bromide (0.5 µg/mL) prior to their identification through photography under UV light. Comparison of the PFGE patterns was performed with Bionumerics software (Applied Maths, Sint-Martens-Latem, Belgium) using the Dice Similarity coefficient. Clusters were defined as DNA patterns sharing more than 85% similarity.

## Results

### Antimicrobial susceptibility

Of the 62 *S. enterica* Typhimurium isolates, 59 (95.2%) were resistant to at least three antimicrobial agents, while only 2 (3.2%) were susceptible to all antimicrobial agents tested. *S. enterica* Typhimurium isolates tested were most commonly resistant to ampicillin (90.3%, 56/62), followed by tetracycline (80.6%, 50/62), trimethoprim/sulfamethoxazole (74.2%, 46/62) and chloramphenicol (66.1%, 41/62). Seventeen (27.4%, 17/62) and 8 (8/62, 12.9%) isolates were resistant to cefotaxime (MIC range from 64 to >256 µg/mL) and ceftazidime (MIC range from 32 to >256 µg/mL), respectively. All ceftazidime-resistant isolates were also resistant to cefotaxime. Twelve isolates (19.4%, 12/62) were highly resistant to ciprofloxacin (MIC≥4 µg/mL). The frequencies of resistance and intermediate to other antimicrobial agents among *S. enterica* Typhimurium isolates were as follows: cefoxitin, 8.1% (5/62) and 1.6% (1/62); aztreonam, 16.1% (10/62) and 12.9% (8/62); cefaclor, 35.5% (22/62) and 0; ampicillin/sulbactam, 51.6% (32/62) and 25.8% (16/62); piperacillin/tazobactam, 19.4% (12/62) and 51.6% (32/62); amikacin, 17.7% (11/62) and 41.9% (26/62); gentamicin, 51.6% (32/62) and 6.5% (4/62); and tobramycin, 56.5% (35/62) and 17.7% (11/62). All isolates were susceptible to imipenem and meropenem.

### β-lactam resistance genes

**Figure 1:**
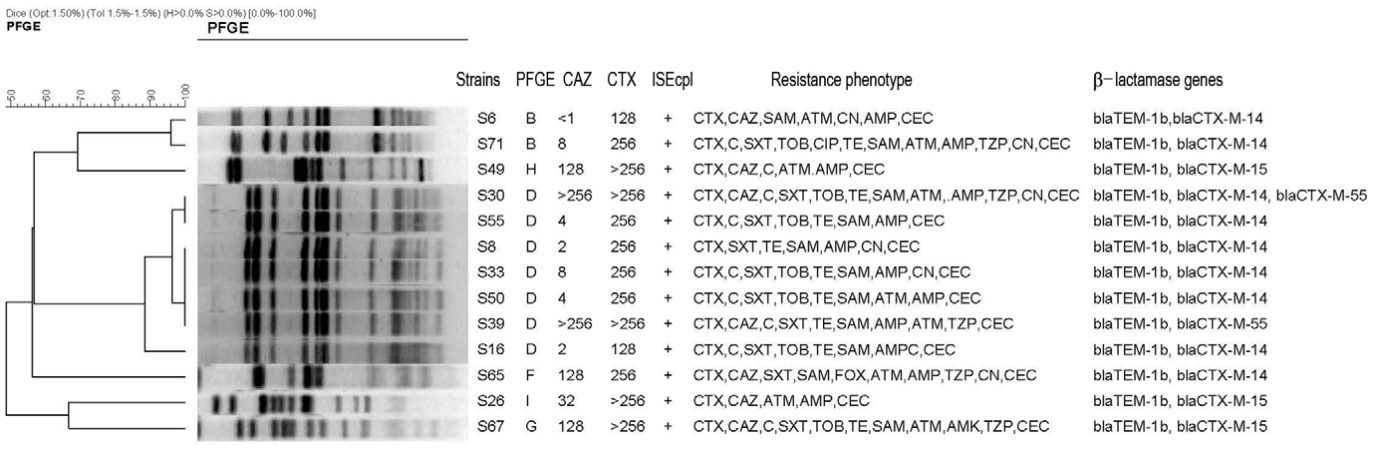
Characteristics of *S. enterica* Typhimurium isolates producing ESBLs. CTX: cefotaxime; CAZ: ceftazidime; SXT: trimethoprim/sulfamethoxazole; TE: tetracycline; SAM: ampicillin plus sulbactam; AMP: ampicillin; CEC: cefaclor; TOB: tobramycin; CN: gentamicin; AMK: amikacin; TZP: piperacillin plus tazobactam; C: chloramphenicol.

Forty-nine (79.0%) of 62 *S. enterica* Typhimurium isolates were positive for *bla*_TEM_ and resistant to ampicillin. All *bla*_TEM_ amplicons were confirmed as *bla*_TEM-1b_ by DNA sequencing. Thirteen *S. enterica* Typhimurium isolates (21.0%, 13/62) were positive for *bla*_CTX-M-1-group_ and *bla*_CTX-M-9-group_ genes (8 for *bla*_CTX-M-14_, 3 for *bla*_CTX-M-15_, 1 for *bla*_CTX-M-55_, and 1 for both *bla*_CTX-M-14_ and *bla*_CTX-M-55_). Characteristics of *S. enterica* Typhimurium isolates producing ESBLs were showed in Figure 1.

Among the 8 CTX-M-14- producing isolates with MICs ranging from 128 to 256 µg/mL for cefotaxime, only one was highly resistant to ceftazidime (128 µg/mL) whereas the other 7 exhibited reduced susceptibility to ceftazidime (2–8 µg/mL). Three CTX-M-15- producing isolates with MICs ranging from 32 to 128 µg/mL for ceftazidime were highly resistant to cefotaxime (MIC>256 µg/mL). Two isolates harboring *bla*_CTX-M-55_ were highly resistant to cefotaxime and ceftazidime (MICs>256 µg/mL). All isolates harboring *bla*_CTX-M_ genes were positive for *IS*Ecp1. Analysis of the flanking regions of *bla*_CTX-M_ genes showed that the insertion sequence *IS*Ecp1 was located 48 bp, 45 bp or 42 bp upstream from *bla*_CTX-M-15_ (accession number GQ330540), *bla*_CTX-M-55_ (accession number GQ456157) or *bla*_CTX-M-14_ (accession number GQ385325), respectively.

### Transfer of antimicrobial resistance genes

Extended-spectrum cephalosporin resistance could be transferred by conjugation from seven ESBL-producing donors (two isolates harboring *bla*_CTX-M-15_, six harboring *bla*_CTX-M-14_, and one harboring both *bla*_CTX-M-14_ and *bla*_CTX-M-55_). Seven cephalosporin resistance transconjugants were resistant to chloramphenicol and trimethoprim/sulfamethoxazole and harbored *bla*_TEM-1b_.

### PFGE

Ten different PFGE clusters were identified among the 62 *S. enterica* Typhimurium isolates. PFGE types A, B, and D accounted for 19.4% (12/62), 9.7% (6/62) and 50% (31/62) of these isolates, respectively. Thirteen ESBLs-producing isolates distributed in six FPGE clusters listed in Figure 1. Of the 8 CTX-M-14-producing *S. enterica* Typhimurium isolates, 5, 2 and 1 belonged to PFGE type D, B and I, respectively. Three CTX-M-15-producing isolates belonged to three different PFGE types (F, G and H). One CTX-M-55-producing isolate and both CTX-M-14 and CTX-M-55-producing isolates belonged to PFGE type D.

## Discussion

The majority of *S. enterica* Typhimurium isolates in our study were resistant to multiple antimicrobial agents, indicating that fewer antibiotics may be useful for treating *S. enterica* Typhimurium infections. Carbapenems exhibit high antimicrobial activity against *S. enterica* Typhimurium isolates *in vitro* in our studies. The CLSI recommends that for fecal isolates of *Salmonella* and *Shigella spp.*, only ampicillin, fluoroquinolone, and trimethoprim/sulfamethoxazole sensitivities should be reported routinely, whereas for extraintestinal isolates of *Salmonella spp.*, only sensitivities to chloramphenicol and third-generation cephalosporins should be reported [13]. However, *S. enterica* Typhimurium resistance rates for ampicillin, trimethoprim/sulfamethoxazole and chloramphenicol were very high (more than 60%) in our studies. In addition, although no patient had received fluoroquinolones prior to isolation of *Salmonella spp.* from their stool, 48% and 19% of *S. Typhimurium* isolates exhibited low- or high-level resistance to ciprofloxacin, respectively. Therefore, our data suggest that ampicillin, trimethoprim/sulfamethoxazole, chloramphenicol and fluoroquinolones should be used with caution for the treatment of *S. enterica* Typhimurium infections in the pediatric population. ESBL- and AmpC-mediated resistance has been reported for nontyphoidal *Salmonella* isolates in many geographic areas [5], [17], [18], [19]. However, reported resistance of nontyphoidal *Salmonella* isolates to broad-spectrum cephalosporins remains low [12], [20], [21]. In the study conducted in Wuhan, China using clinical samples from children ages 0–3 years, only 7 of 221 (3.2%) *S. enterica* isolates were resistant to ceftriaxone (10). In another study from the Henan province, only 2.1% of *S. enterica* Typhimurium patient isolates were resistant to ceftiofur over a 2-year timeframe [11], [12]. Relative to these studies, the rates of resistance to broad-spectrum cephalosporins for *S. enterica* Typhimurium isolates in our study was very high.

Broad-spectrum cephalosporins are commonly used to treat serious *Salmonella* infections. ESBLs are the predominant cause of resistance to broad- spectrum cephalosporins in Enterobacteriaceae, particularly *E. coli* and *Klebsiella spp*. Therefore, understanding patterns and mechanisms for interspecies and intraspecies transfer of ESBLs is of great interest. The TEM-, SHV- and CTX-M-type ESBLs are the most widely distributed worldwide. Published reports indicate that CTX-M-type ESBLs are the most prevalent in China [22], [23]. *bla*_CTX-M-14_ and *bla*_CTX-M-15_ genes have been identified in *S. enterica* Typhimurium isolates from many areas including China [4], . In the present study, CTX-M-type ESBLs, mainly CTX-M-14, were detected in *S. enterica* Typhimurium isolates, indicating that CTX-M-type ESBLs were the predominant cause of resistance to broad-spectrum cephalosporins for *S. enterica* Typhimurium isolates in our study. The mobile genetic element *IS*Ecp1, a single copy insertion sequence responsible for mobilization of *bla* genes and identified upstream of several *bla*_CTX-M_ genes carried by *E. coli* and *Klebsiella spp* isolates, was found in association with all of our isolates. We speculate that the *S. enterica* Typhimurium isolates in China could have acquired the *bla*_CTX-M_ genes from *E. coli* and *Klebsiella spp* in the community, and that subsequent spread of these genes among *S. enterica* Typhimurium isolates may have occurred. Future epidemiologic studies may confirm whether these genes were transmitted in this or perhaps other sequences, and the potential clinical implications of ESBL-mediated resistance for gastrointestinal and/or invasive infections caused by *S. Typhimurium*.

*bla*_CTX-M-55_, which increases the catalytic efficiency against ceftazidime, is a variant of the *bla*_CTX-M-15_ gene and previously found only in *E. coli* and *K. pneumonia spp.* in China and Thailand [25], [26], [27]. Interestingly, we found that two *S. enterica* Typhimurium isolates with resistance to both cefotaxime and ceftazidine harbored *bla*_CTX-M-55_, and one of these harbored both *bla*_CTX-M-14_ and *bla*_CTX-M-55_. To our knowledge, this is the first report of *bla*_CTX-M-55_ in *Salmonella spp*. Recently, 24 (24.5%) of 98 *E. coli* isolates recovered from pets in South China were found to harbor *bla*_CTX-M-55_, among which 6 isolates harbored both *bla*_CTX-M-14_ and *bla*_CTX-M-55_ [25]. The partial DNA sequence of *IS*Ecp1 found 45 bp upstream of *bla*_CTX-M-55_ in the single isolate in our study harboring both *bla*_CTX-M-14_ and *bla*_CTX-M-55_ was 100% identical to the corresponding sequences of *bla*_CTX-M-55_ genes harbored by *E. coli* isolates from the pets in this study. We speculate that *E. coli* which either colonize or cause clinically apparent infection in pets may serve as a reservoir for *bla*_CTX-M-14_ and *bla*_CTX-M-55_ genes and, ultimately, cause clinically apparent infections in pediatric patients. Confirmation of this hypothesis, and the development of subsequent clinical strategies for limited these infections, would require additional comprehensive surveillance efforts among communities in China and elsewhere.

In our study, two main clusters (PFGE types A and D) were found, indicating that gastrointestinal infections in children were caused mainly by clonally related *S. enterica* Typhimurium isolates and clonal spread was responsible for the dissemination of *S. enterica* Typhimurium. Because 5 isolates harboring *bla*_CTX-M-14_ genes and two isolates harboring *bla*_CTX-M-55_ genes belonged to the same PFGE cluster (D), the spread of *bla*_CTX-M-14_ and *bla*_CTX-M-55_ genes was also associated with clonal spread.

In conclusion, the present study demonstrates significant multi-drug resistance among *S.enterica* Typhimurium within a pediatric population in China, including a higher prevalence of broad-spectrum cephalosporin resistance and expression of *bla*_CTX-M_ genes among *S. enterica* Typhimurium isolates than what has previously been reported. Moreover, the present study is the first report of the presence of *bla*_CTX-M-55_ genes in *Salmonella spp*. Finally, our data suggest that clonal spread is responsible for the dissemination of *S. enterica* Typhimurium isolates and *bla*_CTX-M-14_ genes in this population.
